# Extracellular Vesicle‐Mediated Communication Between Anterior Cruciate Ligament and Bone Marrow Cells Modulates Hamstring Tenocyte Behavior and Apoptosis

**DOI:** 10.1002/kjm2.70176

**Published:** 2026-01-24

**Authors:** Hon‐Lok Lo, Shih‐Hao Huang, Ting‐Hsuan Dai, Shun‐Cheng Wu, Cheng‐Jung Ho, Cheng‐Chang Lu

**Affiliations:** ^1^ Department of Orthopedics Kaohsiung Medical University Hospital Kaohsiung Taiwan; ^2^ Regenerative Medicine and Cell Therapy Research Center Kaohsiung Medical University Kaohsiung Taiwan; ^3^ Orthopaedic Research Center Kaohsiung Medical University Kaohsiung Taiwan; ^4^ Ph.D. Program in Biomedical Engineering, College of Medicine Kaohsiung Medical University Kaohsiung Taiwan; ^5^ Department of Orthopedics, School of Medicine, College of Medicine Kaohsiung Medical University Kaohsiung Taiwan; ^6^ Department of Orthopedics, Kaohsiung Municipal Siaogang Hospital Kaohsiung Medical University Kaohsiung Taiwan

**Keywords:** anterior cruciate ligament, apoptosis, bone marrow stromal cells, extracellular vesicles, hamstring graft

## Abstract

Following anterior cruciate ligament (ACL) reconstruction, enhancing hamstring tenocyte activity and minimizing apoptosis are critical for preventing graft failure and promoting ligamentization. This study investigated the therapeutic potential of extracellular vesicles (EVs) derived from a coculture of ACL remnant cells and bone marrow stromal cells (BMSCs), termed coculture‐EV, in comparison with EVs from BMSC monoculture (BMSC‐EV). We hypothesized that coculture‐EVs could enhance tenocyte activity and reduce apoptosis, with greater effects than BMSC‐EVs. ACL remnants, bone marrow, and hamstring tendons were harvested from rabbits 4 weeks post‐ACL transection, and the cultured cells were used for coculture and subsequent experiments. EVs were isolated by ultracentrifugation and characterized by nanoparticle size analysis, electron microscopy, and EV‐specific markers. Hamstring tenocytes treated with coculture‐EVs exhibited significantly improved viability, proliferation (EdU assay), and migration (scratch and transwell assays), along with increased expression of genes related to collagen synthesis, transforming growth factor beta (TGF‐β), and vascular endothelial growth factor (VEGF). Importantly, coculture‐EV treatment more effectively suppressed both intrinsic and extrinsic apoptotic pathways. Coculture‐EV treatment reduced early apoptosis in tenocytes by 54.5%, whereas BMSC‐EV produced a 21.1% reduction. These findings suggest that EVs from ACL/BMSC coculture possess superior bioactivity compared with BMSC‐derived EVs alone. Coculture‐EV enhances tenocyte function and survival, indicating its potential as a cell‐free therapeutic strategy to promote hamstring graft maturation and improve outcomes after ACL reconstruction.

## 1 Introduction

Anterior cruciate ligament (ACL) injuries are among the most common sports‐related injuries of the knee joint and often require surgical reconstruction to restore knee stability [1, 2]. During ACL reconstruction, a tendon graft is implanted and undergoes a series of biological processes, including tenocyte apoptosis, revascularization, cellular proliferation, remodeling, and maturation [3, 4]. Minimizing apoptosis of grafted tenocytes is critical for enhancing tendon graft maturation, preventing graft failure, and ensuring successful ACL reconstruction. Preservation of ACL remnants has been proposed as a strategy to improve graft maturation after surgery [5, 6]. Our previous studies have shown that ACL remnants contain stem/progenitor cells and exert beneficial paracrine effects on the surrounding microenvironment, contributing to reduced apoptosis and improved graft maturation [7, 8, 9, 10]. However, although these studies suggest the involvement of paracrine mechanisms, they did not isolate extracellular vesicles (EVs) or evaluate their specific functional roles. In particular, the distinct effects of bone marrow stromal cell (BMSC)‐derived EVs versus coculture‐derived EVs on hamstring tenocytes—which are directly relevant to ACL graft biology—remain unclear.

During ACL reconstruction surgery, bone tunnels are drilled to allow passage of the hamstring graft. In the context of ACL remnant preservation, the implanted hamstring graft comes into contact with both bone marrow released from the tunnels and the ACL remnant. However, the combined effect of the ACL remnant and bone marrow on the implanted hamstring graft is not well understood. To investigate the interaction between the ACL remnant and bone marrow and its effect on the hamstring tendon, Lu et al. designed a direct coculture system to simulate the coexistence of the ACL remnant and bone marrow during ACL reconstruction [8, 11]. They found that the coculture medium enhanced the activity of ACL remnant cells and BMSCs, reduced apoptosis, and promoted tenocyte activity. Furthermore, the coculture medium, generated through interactions between ACL cells and BMSCs, was more effective than BMSC monoculture medium. However, the primary components responsible for the effects of the coculture medium remain unclear.

Extracellular vesicles (EVs) are membrane‐bound particles secreted by cells into the extracellular space that carry a diverse cargo of proteins, lipids, RNAs, and other bioactive molecules [12, 13]. By mediating intercellular communication, EVs influence a broad range of physiological processes, including immune modulation, signal transduction, and tissue regeneration. Owing to the synergistic effects of their molecular contents, EVs have emerged as a promising therapeutic option for tissue repair [14, 15, 16]. Given this, it is reasonable to assume that EVs are present in the coculture medium derived from ACL remnant cells and BMSCs. Lu et al. [11] demonstrated that removal of EVs from the coculture medium significantly diminished its ability to enhance the activity of ACL cells and BMSCs. Conversely, treatment with EVs isolated from the coculture medium (coculture‐EV) effectively restored and promoted the activity of both ACL cells and BMSCs. These findings suggest that EVs are the primary functional constituents of the ACL remnant cell/BMSC coculture medium and play a central role in mediating the paracrine interaction between these two cell types. Based on Lu's studies, such EVs may deliver miRNAs and growth factor‐related proteins that modulate downstream survival pathways, suppress intrinsic apoptotic signaling, and promote the activity of recipient musculoskeletal cells, including tenocytes. Nonetheless, the specific effects of coculture‐derived EVs on hamstring tenocytes remain to be fully elucidated and warrant further investigation.

This study aims to assess the effects of coculture‐EV on hamstring tenocytes, in comparison with EVs derived from BMSC monoculture medium (BMSC‐EV), with a focus on their influence on cell activity and apoptotic pathways. We hypothesize that coculture‐EVs are more effective than BMSC‐EVs in enhancing hamstring tenocyte activity and reducing apoptosis, representing a novel strategy for improving tendon graft maturation in ACL reconstruction. Our study addresses this gap by directly isolating and characterizing both EV populations and systematically evaluating their effects on tenocyte viability, migration, and intrinsic apoptotic signaling. This study provides the first evidence clarifying the distinct EV‐mediated mechanisms that may contribute to improved tendon graft maturation.

## 2 Materials and Methods

### 2.1 Study Design and Animal Use

In this study, tissues from the ACL remnant (4 weeks post‐ACL transection), bone marrow (from the iliac crest), and hamstring tendon were collected from six skeletally mature male New Zealand rabbits, each weighing 2.5–3.0 kg. All animal procedures, including tissue harvesting and cell culture, followed the methods outlined by Lu et al. [8]. Cells at the third passage, including ACL remnant cells, BMSCs, and hamstring tenocytes, were used for subsequent experiments. The study was approved by the Institutional Animal Care and Use Committee at Kaohsiung Medical University (approval number KMU‐107193). The use of six rabbits was based on our previous study [9], in which a pilot analysis using G*Power 3.1.9.7 (Franz Faul) indicated that six animals were sufficient to detect meaningful differences while adhering to the 3R principles (replacement, reduction, and refinement).

### 2.2 Tissue Collection and Cell Culture

ACL remnant cells (1.5 × 10^5^ cells) and BMSCs (1.5 × 10^5^ cells) were directly cocultured in 10 mL medium consisting of low‐glucose Dulbecco's Modified Eagle Medium (DMEM), 10% exosome‐depleted fetal bovine serum (FBS; Cat. No. A2720801, Gibco, Thermo Fisher Scientific, Waltham, MA, USA), and 1% antibiotics (penicillin/streptomycin) in a 10‐cm culture dish at 37°C with 5% CO_2_. For the control group, BMSCs (3 × 10^5^ cells) were cultured under the same conditions using the same medium. Culture media from both the BMSC monoculture and coculture groups were collected every 2 days for 6 days [11].

### 2.3 EV Isolation and Characterization

EVs were isolated from both the BMSC monoculture medium and the ACL/BMSC coculture medium using a sequential ultracentrifugation method, as described by Lu et al. [11] Briefly, 500 mL of culture medium was centrifuged at 20,000 × *g* for 30 min at 4°C to remove large cell debris. The supernatant was then collected and centrifuged at 120,000 × *g* for 90 min at 4°C to pellet membrane‐bound vesicles. After discarding the supernatant, the pellet was washed with 10 mL of phosphate‐buffered saline (PBS) and subjected to a second centrifugation at 120,000 × g for 90 min at 4°C. Following removal of the supernatant, the pellet containing EVs was resuspended in 1 mL of PBS and filtered through a 0.22‐μm filter membrane.

The isolated EVs were analyzed for size and morphology using nanoparticle tracking analysis (NTA) and electron microscopy. Western blotting was performed to detect positive EV markers (CD9, CD63, CD81, Alix, and TSG101) and a negative marker (α‐tubulin), following the methods described by Lu et al. [11].

### 2.4 Experimental Procedures

Third‐passage hamstring tenocytes were treated with either BMSC‐EV or coculture‐EV, while hamstring tenocytes without EV treatment served as the control group. Each independent experiment included six samples per group, and all measurements were performed in triplicate. These groups were subsequently evaluated in the following experiments.

#### 2.4.1 Cell Viability

The viability of hamstring tenocytes treated with BMSC‐EV, coculture‐EV, or a non‐EV control was evaluated using the Cell Counting Kit‐8 (CCK‐8) (Cat. No. KTA1020, Abbkine), according to the manufacturer's guidelines. Cells were seeded in 96‐well plates at a density of 5 × 10^3^ cells per well in 100 μL of low‐glucose DMEM supplemented with 10% FBS and 1% penicillin/streptomycin, and incubated overnight at 37°C with 5% CO_2_. The following day, BMSC‐EV or coculture‐EV were added at a ratio of 10^10^ particles per 10^4^ cells, with the total well volume adjusted to 180 μL. After 24 h of incubation, 20 μL of CCK‐8 solution was added to each well and incubated for 2 h. Absorbance was measured at 450 nm using a Bio‐Rad Microplate Manager Benchmark Plus Reader (Bio‐Rad Laboratories, Hercules, CA, USA).

#### 2.4.2 Cell Proliferation

Cell proliferation was assessed using the Click‐iT EdU assay kit (Thermo Fisher Scientific) and Ki67 gene expression analysis. Hamstring tenocytes (2 × 10^4^ cells/well) were seeded in 12‐well plates and incubated overnight. The following day, BMSC‐EV or coculture‐EV (10^10^ particles per 10^4^ cells) and 10 μM EdU were added to the culture medium, followed by a 24‐h incubation at 37°C with 5% CO_2_. Cells were then fixed with 4% formaldehyde for 15 min at room temperature, washed with 3% BSA in PBS, and permeabilized with 0.5% Triton X‐100. After additional washes, the Click‐iT reaction cocktail was applied, and the plates were incubated in the dark for 30 min. Nuclei were counterstained with Hoechst 33342, and cell slides were mounted. Proliferation was quantified in five randomly selected fields per sample using ImageJ (NIH). Ki67 gene expression was analyzed as a marker of proliferation, as described in the RT‐PCR section.

#### 2.4.3 Migration

To investigate the effects of BMSC‐EV, coculture‐EV, or non‐EV treatments on hamstring tenocyte migration, scratch and transwell migration assays were performed. For the scratch migration assay, closure of the scratch gap was observed at 12, 24, 36, and 48 h under a microscope (Leica DMI6000B; Leica Microsystems, Germany). The transwell migration assay was conducted using a 6.5‐mm chamber with an 8 μm pore size (Cat. No. 3422, Costar, Corning Inc., Corning, NY, USA). After allowing cells to migrate, they were fixed with 4% formaldehyde for 15 min, washed twice with PBS, and treated with methanol for 10 min. Migrated cells were then stained with 0.1% crystal violet for 30 min. The number of purple‐stained cells was counted under a microscope, and the relative migration rate was calculated by comparing the number of migrated cells in each treatment group with that in the control group.

#### 2.4.4 Gene Expression and Immunofluorescence Staining (RT‐PCR)

Total RNA was isolated from hamstring tenocytes treated with BMSC‐EV, coculture‐EV, or non‐EV using RNAzol reagent (Cat. No. RN‐190, Molecular Research Center, Cincinnati, OH, USA). Approximately 2 μg of total RNA was reverse‐transcribed using the Maxima First Strand cDNA Synthesis Kit (Cat. No. K1642, Thermo Fisher Scientific, Waltham, MA, USA), according to the manufacturer's guidelines. The primers, cycling conditions, and quantification methods were as previously described [8]. The list of primers used is provided in Table 1.

**TABLE 1:** The primers of complementary DNA sequences.

Gene name	Amplicon size (bp)	Primer sequence	Accession No.
Rb‐COL‐1	73	Forward 5′‐TTCTGCAGGGCTCCAATGA‐3′ Reverse 5′‐TCGACAAGAACAGTGTAAGTGAACCT‐3′	NM_001195668.1
Rb‐COL‐3	92	Forward 5′‐CCTGAAGCCCCAGCAGAA‐3′ Reverse 5′‐AACAGAAATTTAGTTGGTCACTTGTACTG‐3′	XM_002712333.3
Rb‐VEGF	122	Forward 5′‐ATCATGCGGATCAAACCTCA‐3′ Reverse 5′‐CAAGGCCCACAGGGATTTTC‐3′	XM_020912728.1
Rb‐TGFβ	140	Forward 5′‐CAGTGGAAAGACCCCACATCTC‐3′ Reverse 5′‐GACGCAGGCAGCAATTATCC‐3′	XM_008268050
Rb‐Ki67	232	Forward 5′‐GTCACCGAGAGGCAGAGAAC‐3′ Reverse 5′‐TTTGCCCTTCTTCCACATTC‐3′	XM_008251084.2
Rb‐SCX	165	Forward 5′‐CAGCGGCACACGGCGAAC‐3′ Reverse 5′‐CGTTGCCCAGGTGCGAGATG‐3′	BK000280
Rb‐TNC	78	Forward 5′‐CAGAAGCCTTGGCCATGTG‐3′ Reverse 5′‐GCACTCTCTCCCCTGTGTAGGA‐3′	XM_017350093
Rb‐TNMD	122	Forward 5′‐CCATGCTGGATGAGAGAGGTT‐3′ Reverse 5′‐CCGTCCTCCTTGGTAGCAGT‐3′	NM_001109818.1
Rb‐Caspase 3	129	Forward 5′‐GCTGGACAGTGGCATCGAGA‐3′ Reverse 5′‐TCCGAATTTCGCCAGGAATAGTAA‐3′	NM_001082117.1
Rb‐Caspase 8	179	Forward 5′‐ATGCAGAGGCTTTGAGCAAT3′ Reverse 5′‐GCCATAGATGATGCCCTTGT‐3′	XM_017343029.1
Rb‐Caspase 9	133	Forward 5′‐CTGTTTCCGAGCGAGGGATT‐3′ Reverse 5′‐CGCAGGAAGGTTTTGGGGTA‐3′	XM_008249762.2
RB‐Bax	250	Forward 5′‐GTCGCCCTGTTTTACTTTGC‐3′ Reverse 5′‐CTCAGCCCATCTTCTTCCAG‐3′	XM_002723696.3
Rb‐Bcl‐2	221	Forward 5′‐GATTGTGGCCTTCTTTGAGTTC‐3′ Reverse 5′‐AAGTCTTCAGAGACACCCAGGA‐3′	XM_008261439.2
Rb‐Bcl‐xL	150	Forward 5′‐CACCCGGAGAACCACTACAT‐3′ Reverse 5′‐AGCTCTGGGGGAAATTTTGT‐3′	XM_008256141.2
Rb‐Fas	442	Forward 5′‐CAAACCAGCAACACCAAATGC‐3′ Reverse 5′‐CCGCAAGAGCACAAAGATTAG‐3′	XM_008269902.2
Rb‐GAPDH	103	Forward 5′‐AGTGACACCCACTCCTCCAC‐3′ Reverse 5′‐TGCTGTAGCCAAATTCGTTG‐3′	NM_001082253

Abbreviations: Bax, Bcl2‐associated X protein; Bcl‐xL, B‐cell lymphoma‐extralarge; Bcl2, B‐cell lymphoma 2; COL‐1, Collagen‐I; COL‐3, Collagen‐III; Fas, Fas cell surface death receptor; GAPDH, glyceraldehyde‐3‐phosphate dehydrogenase; ki67, marker of proliferation Ki‐67; Rb, rabbit; SCX, scleraxis; TGFβ, transforming growth factor beta 1; TNC, tenascin D; TNMD, tenomodulin; VEGF, vascular endothelial growth factor.

Hamstring tenocytes treated with BMSC‐EV, coculture‐EV, or non‐EV were evaluated for immunofluorescence expression of COL‐I and COL‐III, TGF‐β, and VEGF. The primary antibodies, fluorescent secondary antibodies, and staining procedures were performed as previously described [8]. The primary antibodies used were anti‐COL‐I (1:200, Cat. No. ARG21965, Arigo Biotechnology, Hsinchu City, Taiwan), anti‐COL‐III (1:200, Cat. No. ARG20786, Arigo Biotechnology), anti‐TGF‐β (1:200, Cat. No. ARG10002, Arigo Biotechnology), and anti‐VEGF (1:200, Cat. No. ARG10513, Arigo Biotechnology). The secondary fluorescent antibodies employed were CoraLite594‐conjugated donkey anti‐mouse IgG (H + L) (1:250, Cat. No. SA00013‐7, Proteintech, Chicago, IL, USA) and goat anti‐rabbit IgG (H + L)‐TAMRA (1:250, Cat. No. LDG0047YE, Leadgene Biomedical Inc., Hsinchu, Taiwan).

#### 2.4.5 Apoptosis Analysis

To assess the effects of BMSC‐EV and coculture‐EV on hamstring tenocyte apoptosis, apoptosis was evaluated using flow cytometry with Annexin V‐fluorescein isothiocyanate/propidium iodide (PI) staining, along with PCR analysis of gene expression associated with the intrinsic apoptotic pathway (*Bax*, *Bcl‐2*, *Caspase 9*, and *Bcl‐xL*) and the extrinsic apoptotic pathway (*Fas*, *Caspase 3*, and *Caspase 8*). The primers used for apoptosis‐related genes are listed in Table 1.

##### 2.4.5.1 Annexin V/PI Staining

Apoptosis of hamstring tenocytes treated with BMSC‐EV, coculture‐EV, or non‐EV was assessed using the Annexin V/PI assay by flow cytometry (FC500; Beckman Coulter, USA). Tenocytes were stained with Annexin V‐FITC and PI according to the manufacturer's protocol (Cat. No. 32113; Leadgene Biomedical, Taiwan). Briefly, 1–5 × 10^5^ tenocytes were detached using 1× trypsin, washed with PBS, and pelleted by centrifugation at 300 × *g* for 5 min. The cell pellet was resuspended in 500 μL of 1× Binding Buffer (Leadgene Biomedical, Taiwan). Subsequently, 5 μL of Annexin V‐FAM (carboxyfluorescein) and 5 μL of PI were added, and the cells were incubated in the dark for 15–20 min at room temperature. After incubation, the cells were kept on ice and analyzed by flow cytometry using appropriate filters for fluorescein isothiocyanate (FITC), corresponding to Annexin V‐FAM. Early apoptotic cells were identified as Annexin V positive and PI‐negative, whereas late apoptotic cells were double positive for Annexin V and PI. Necrotic cells were characterized as Annexin V‐negative and PI‐positive.

### 2.5 Statistical Analysis

Data were analyzed using one‐way analysis of variance (ANOVA), followed by Tukey's post hoc test for multiple comparisons, using SPSS software version 20 (IBM, USA). All data are presented as the mean ± standard deviation (SD) based on three measurements. In this study, values for the control group were normalized to 1, and values for the experimental groups are expressed relative to the control. A *p*‐value < 0.05 was considered statistically significant.

## 3 Results

### 3.1 EV Characterization

**FIGURE 1:**
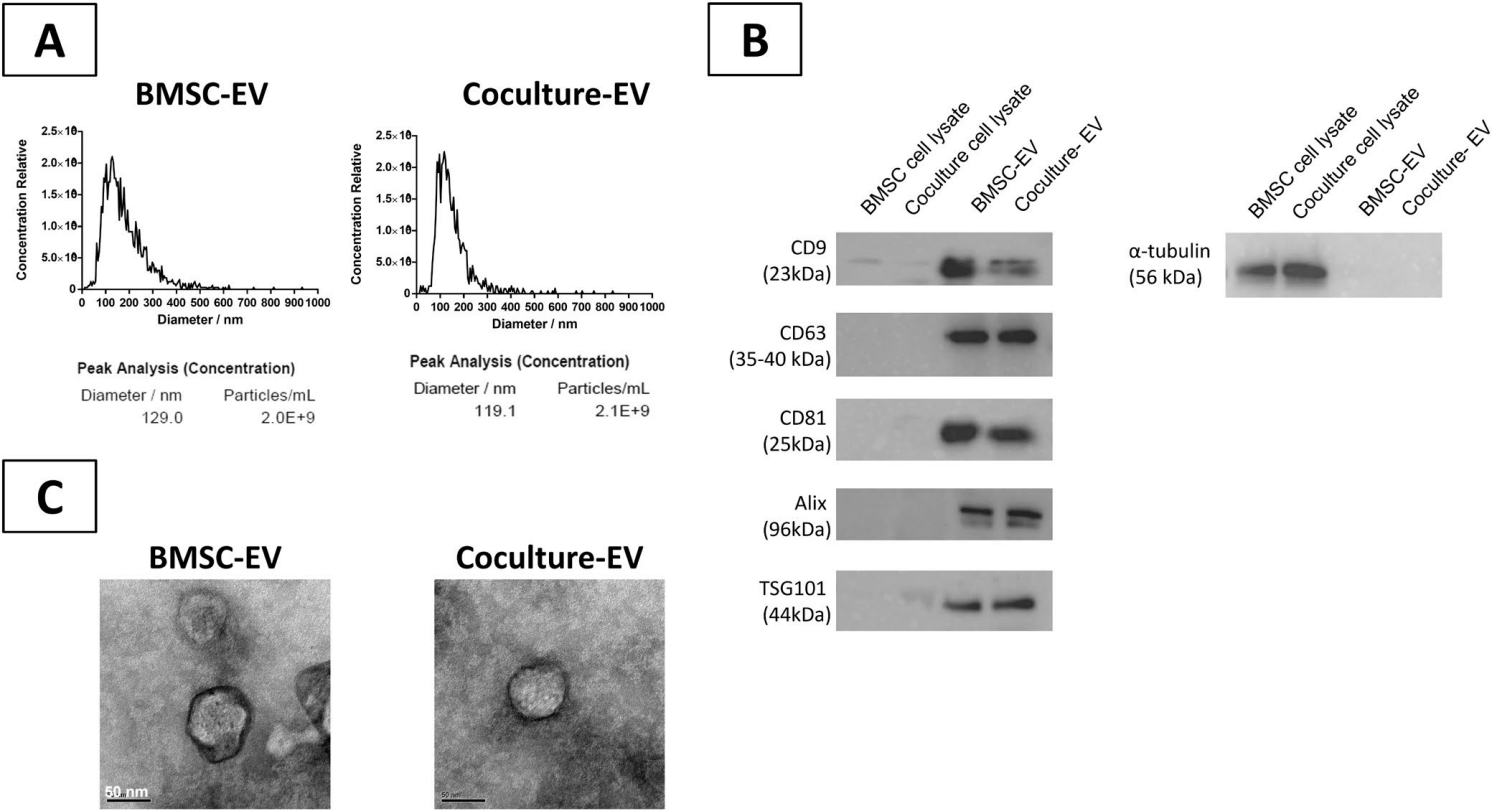
(A) Particle size distribution, (B) biomarker expression levels, and (C) transmission electron microscopy images of extracellular vesicles (EVs) isolated from bone marrow‐derived mesenchymal stem cell (BMSC) monoculture (BMSC‐EV) and from the coculture of anterior cruciate ligament (ACL) remnant cells with BMSCs (CM‐EV).

The nanoparticle tracking analysis (NTA) showed that the average diameters of BMSC‐EVs and coculture‐EVs were 129 and 119 nm, with particle concentrations of 2.0 × 10^9^ and 2.1 × 10^9^ per mL, respectively (Figure 1A). Both EV types were positive for EV markers, including CD9, CD63, CD81, Alix, and TSG101, while lacking the negative marker α‐tubulin (Figure 1B). In addition, microscopic images revealed that both BMSC‐EVs and coculture‐EVs exhibited a spherical morphology with a light‐colored hypodense center and a dark‐colored hyperdense periphery (Figure 1C). These results confirm that EVs were successfully isolated from both BMSC monoculture and coculture media, in accordance with the Minimal Information for Studies of Extracellular Vesicles (MISEV) 2014 and 2018 guidelines [17, 18].

### 3.2 Treatment With Coculture‐EV Improved the Viability, Proliferation, Migration, and Gene Expression in Hamstring Tenocytes

**FIGURE 2:**
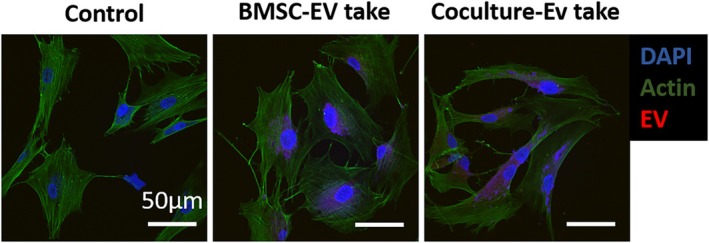
Immunofluorescence images illustrating the uptake and internalization of BMSC‐EV and CM‐EV by hamstring tenocytes.

**FIGURE 3:**
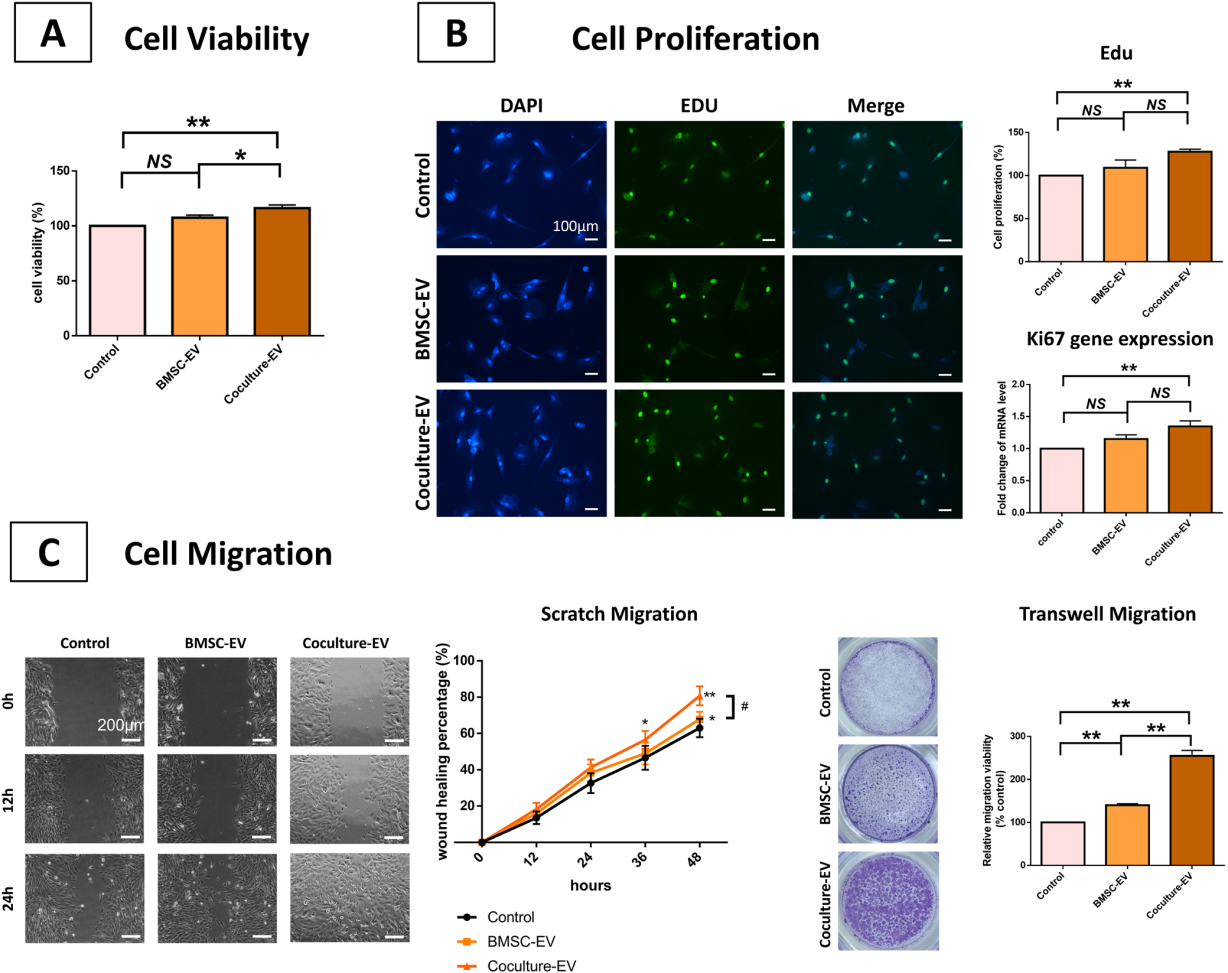
(A) Viability, (B) proliferation, and (C) migration of hamstring tenocytes following treatment with either no extracellular vesicles (control), EVs from BMSC monoculture medium (BMSC‐EV), or EVs from ACL remnant cell/BMSC coculture medium (CM‐EV). *p < 0.05; **p < 0.01.

Both BMSC‐EVs and coculture‐EVs were successfully taken up by hamstring tenocytes (Figure 2). Hamstring tenocytes treated with coculture‐EVs exhibited significantly higher cell viability compared with both the BMSC‐EV‐treated and control groups (Figure 3A). In terms of cell proliferation, as indicated by EdU incorporation and Ki67 gene expression, coculture‐EV‐treated tenocytes showed a marked increase compared with the control group (Figure 3B). Furthermore, coculture‐EV treatment significantly enhanced the migratory capacity of tenocytes, as demonstrated by both scratch wound and transwell migration assays (Figure 3C).

**FIGURE 4:**
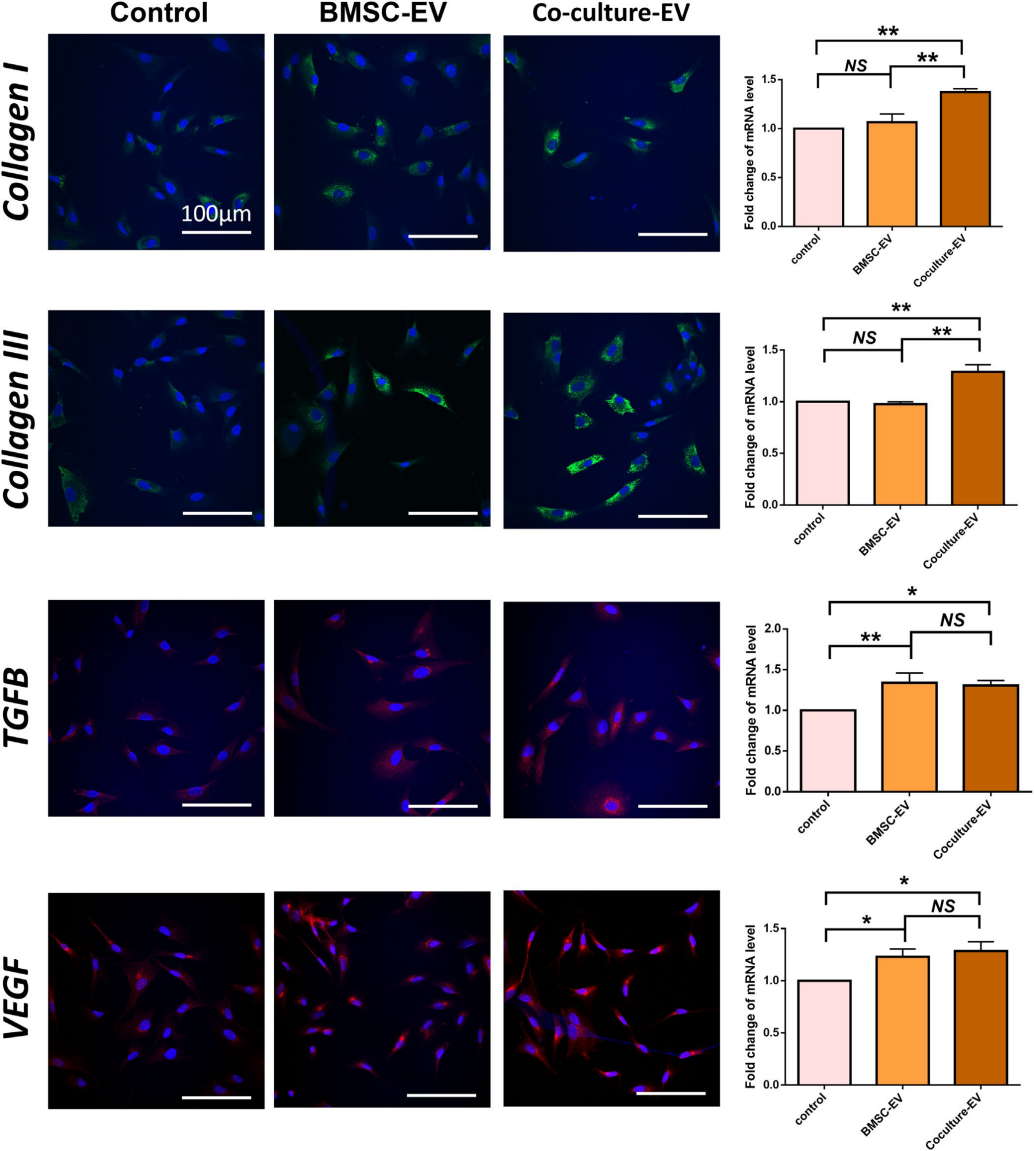
Expression levels of Collagen Type I, Collagen Type III, TGF‐β, and VEGF gene in hamstring tenocytes treated with either no extracellular vesicles (control), EVs derived from BMSC monoculture medium (BMSC‐EV), or EVs from ACL remnant cell/BMSC coculture medium (CM‐EV). *p < 0.05; **p < 0.01.

**FIGURE 5:**
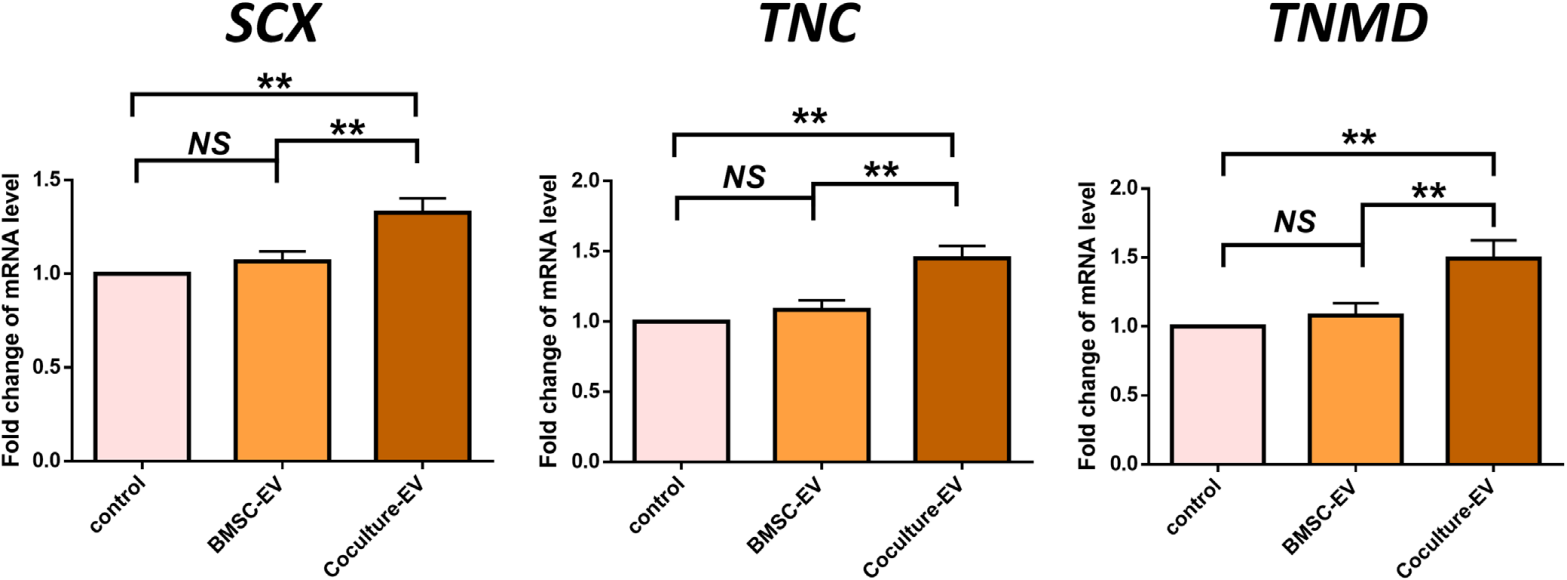
Expression of tenogenic markers (Scx, TNC, and TNMD) in hamstring tenocytes treated with either no extracellular vesicles (control), EVs derived from BMSC monoculture medium (BMSC‐EV), or EVs from ACL remnant cell/BMSC coculture medium (CM‐EV). *p < 0.05; **p < 0.01.

Gene expression analysis revealed that tenocytes treated with coculture‐EVs exhibited significantly higher expression levels of collagen type *I* and *III* compared with both the BMSC‐EV‐treated and control groups. In addition, coculture‐EV treatment resulted in significantly increased expression of *TGFβ* and *VEGF* relative to the control group (Figure 4). Moreover, the expression of tenogenic markers, including *Scx*, *TNC*, and *TNMD*, was markedly higher in coculture‐EV‐treated tenocytes than in those treated with BMSC‐EVs or in the control group (Figure 5).

### 3.3 Coculture‐EV Effectively Attenuated Apoptosis in Hamstring Tenocytes

**FIGURE 6:**
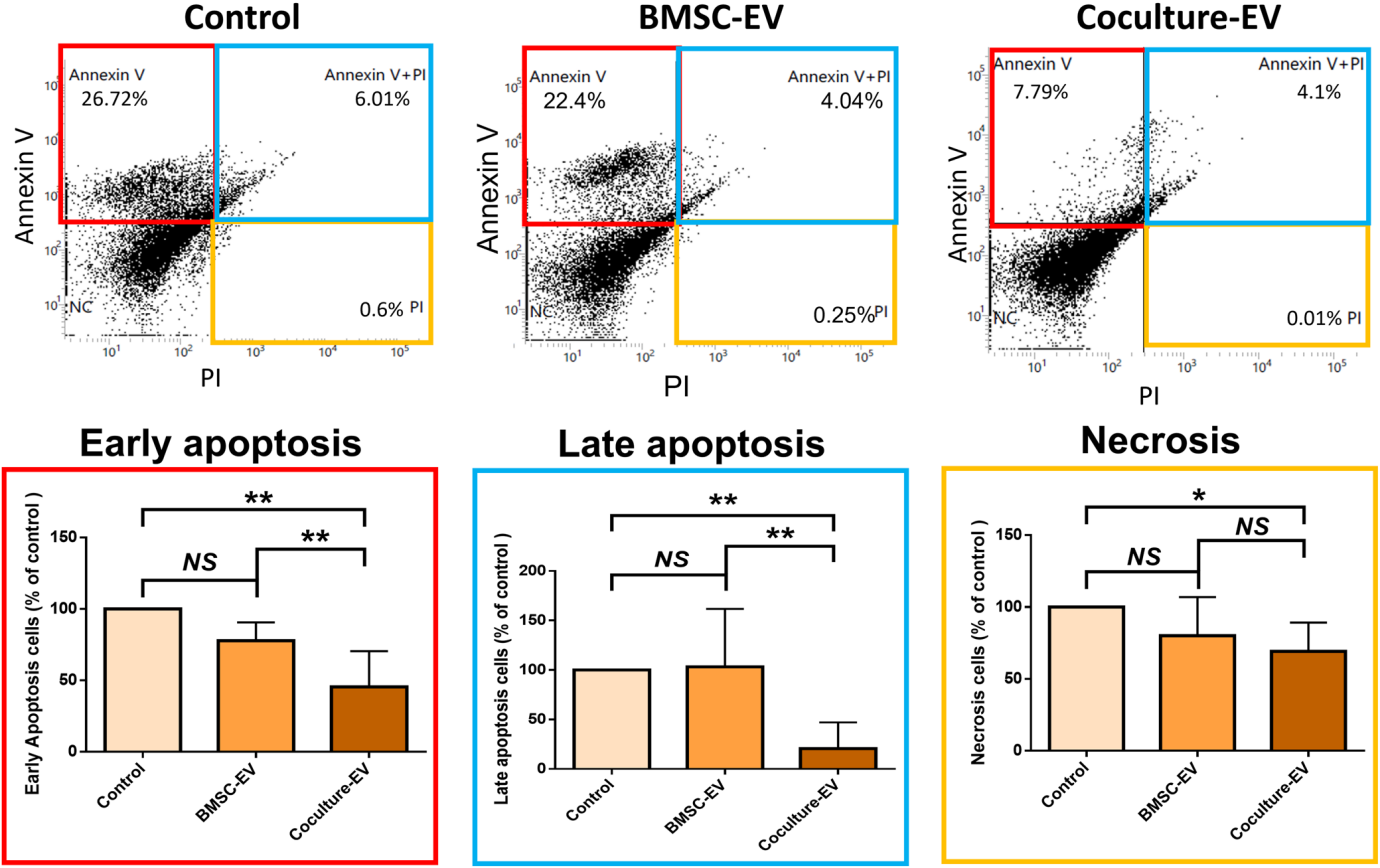
Effects of extracellular vesicles (EVs) on apoptosis regulation in hamstring tenocytes. Tenocytes were treated with either no EVs (control), EVs derived from BMSC monoculture medium (BMSC‐EV), or EVs from ACL remnant cell/BMSC coculture medium (CM‐EV). Apoptotic profiles were evaluated by flow cytometry following Annexin V‐FITC and propidium iodide (PI) staining. Representative dot plots illustrate live cells (Annexin V−/PI−, lower left quadrant), early apoptotic cells (Annexin V+/PI−, upper left), late apoptotic cells (Annexin V+/PI+, upper right), and necrotic cells (Annexin V−/PI+, lower right). *p < 0.05; **p < 0.01. All values are normalized to the control group (set as 1.0 or 100%). Early apoptosis: Control 100%; BMSC‐EV 77.87% ± 12.70%; coculture‐EV 45.49% ± 24.92%. Late apoptosis: Control 100%; BMSC‐EV 103.30% ± 58.48%; coculture‐EV 20.58% ± 26.54%. Necrosis: Control 100%; BMSC‐EV 87.49% ± 22.10%; coculture‐EV 75.11% ± 15.51%.

Flow cytometric analysis using Annexin V/PI staining demonstrated a significantly lower percentage of both early and late apoptotic cells in the coculture‐EV‐treated group compared with the BMSC‐EV‐treated and control groups. In addition, coculture‐EV‐treated tenocytes exhibited a significant reduction in necrotic cells relative to the control group (Figure 6). Compared with the control group, BMSC‐EV reduced early apoptosis by 22.1%, while coculture‐EV produced a 54.5% reduction. For late apoptosis, BMSC‐EV showed no appreciable decrease (−3.3%), whereas coculture‐EV reduced late apoptosis by 79.4%. With respect to necrosis, BMSC‐EV reduced the rate by 12.5%, and coculture‐EV by 24.9%.

**FIGURE 7:**
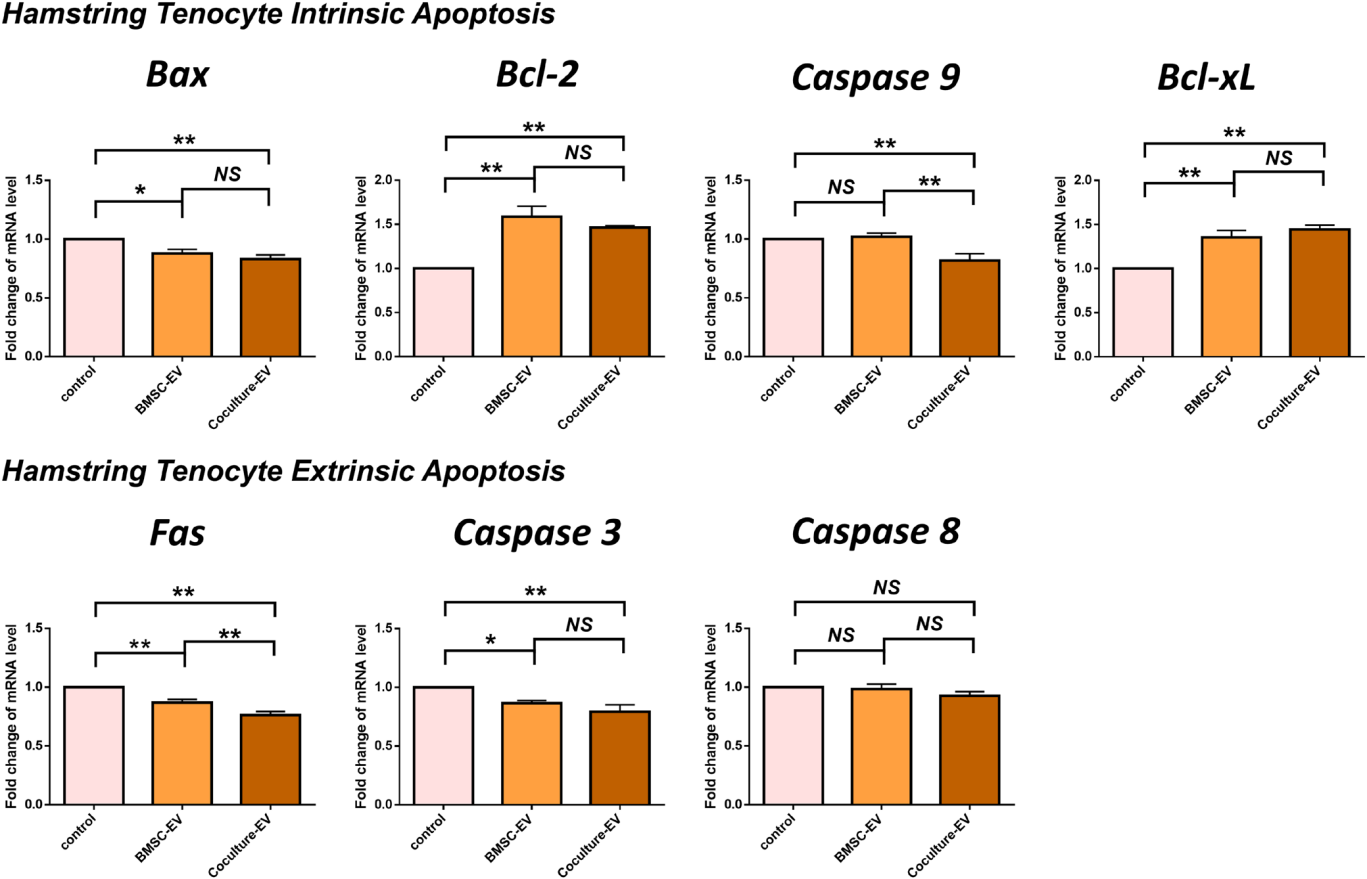
Effects of extracellular vesicles (EVs) on intrinsic and extrinsic apoptosis genes expression in hamstring tenocytes. Tenocytes were treated with either no EVs (control), EVs derived from BMSC monoculture medium (BMSC‐EV), or EVs from ACL remnant cell/BMSC coculture medium (CM‐EV). *p < 0.05; **p < 0.01. Bax, Bcl2‐associated X protein; Bcl2, B‐cell lymphoma 2; Fas, Fas cell surface death receptor.

Coculture‐EV effectively reduced tenocyte apoptosis by modulating both intrinsic and extrinsic apoptotic pathways. The expression of the pro‐apoptotic genes *Bax* and *Caspase 9* was lower in both the BMSC‐EV‐ and coculture‐EV‐treated groups compared with the control, with a greater reduction observed in the coculture‐EV group. In addition, the expression of the anti‐apoptotic genes *Bcl‐2* and *Bcl‐xL* was higher in both the BMSC‐EV and coculture‐EV groups compared with the control. These findings indicate that coculture‐EV exerted the most potent inhibitory effect on the intrinsic apoptotic pathway (Figure 7).

Regarding the extrinsic apoptotic pathway, gene expression of *Fas* and *Caspase 3* was significantly reduced in both the BMSC‐EV and coculture‐EV groups compared with the control, with the coculture‐EV group showing a stronger inhibitory effect. No significant differences in Caspase 8 expression were observed among the control, BMSC‐EV, and coculture‐EV groups (Figure 7).

## 4 Discussion

Enhancement of cell activity and reduction of apoptosis in implanted hamstring tendons and tenocytes are crucial for graft maturation following ACL reconstruction. In this study, EVs derived from the coculture medium of ACL remnant cells and BMSCs significantly improved hamstring tenocyte activity compared with EVs derived from BMSC monoculture or the non‐EV‐treated group (Figure 3). Following coculture‐EV treatment, hamstring tenocytes exhibited marked increases in cell viability, proliferation, and migration, along with upregulation of genes associated with collagen synthesis, *TGF‐β*, and *VEGF* (Figure 4). Furthermore, coculture‐EVs reduced tenocyte apoptosis by modulating both intrinsic and extrinsic apoptotic pathways, demonstrating a strong protective effect on hamstring tenocytes (Figures 6 and 7). These findings support our hypothesis that EV‐mediated communication between ACL remnant cells and BMSCs modulates hamstring tenocyte behavior and apoptosis. Although BMSC‐derived EVs have been widely studied in tendon repair, limited information is available regarding EVs produced in tendon‐related coculture systems. To date, only one report has shown that hypoxic shoulder tenocytes cocultured with adipose‐derived MSCs release exosomes enriched in matrix‐regeneration proteins [19]. Our study expands this limited body of evidence by directly comparing BMSC‐EVs with coculture‐EVs and demonstrating that coculture‐EVs exert distinct and superior effects on tenocyte activity.

Extracellular vesicles derived from mesenchymal stem cells (MSCs) have emerged as important modulators of apoptosis in diverse biological contexts [20, 21, 22]. Phetfong et al. [20] demonstrated that MSC‐EVs inhibited proliferation and induced apoptosis in leukemic cells, suggesting a potential role in cancer suppression. In contrast, Tati et al. [21] reported that MSC‐EVs promoted proliferation and reduced apoptosis in cultured corneal epithelial cells, likely through modulation of Caspase‐3, thereby facilitating corneal tissue repair. Zhang et al. [22] investigated the effects of MSC‐EVs on chondrocytes using immortalized E1‐MYC 16.3 human embryonic stem cell‐derived MSCs and showed that EVs enhanced chondrocyte proliferation and reduced apoptosis via CD73‐mediated adenosine signaling, which activates the AKT and ERK pathways. Consistent with these findings, our study demonstrated that EVs isolated from the coculture medium of ACL cells and BMSCs attenuated both intrinsic and extrinsic apoptotic pathways during early and late phases in hamstring tenocytes, while simultaneously enhancing their proliferation. Collectively, these studies underscore the context‐dependent role of MSC‐derived EVs in apoptosis regulation, which is influenced by the MSC source, recipient cell type, and culture conditions.

The composition and secretion of EVs are significantly influenced by external factors such as culture conditions, microenvironmental cues, and external stimulation [23, 24, 25]. Pulido‐Escribano et al. [23] examined the behavior of mesenchymal stem cells (MSCs) under hypoxic (low‐oxygen) conditions and reported that EVs released in this environment possess greater regenerative potential than those generated under normoxic (normal oxygen) conditions. Similarly, Li et al. [24] investigated the application of low‐intensity pulsed ultrasound (LIPUS) to BMSCs and found that LIPUS at 300 mW/cm^2^ increased EV secretion by 3.66‐fold, while also enhancing the expression of miR‐328‐5p and miR‐487b‐3p within the secreted vesicles. In the present study, we recreated the physiological microenvironment of ACL reconstruction by coculturing ACL cells with BMSCs. This interaction stimulated the release of paracrine EVs that enhanced the viability and functional activity of hamstring tenocytes.

During the initial stages of graft maturation, hamstring tendon grafts often undergo substantial tenocyte apoptosis, which weakens structural integrity and impairs healing, thereby compromising long‐term graft success. Clinically, improving the biological environment of the tendon graft is critical for reducing graft failure and promoting faster recovery. In this context, EVs have emerged as a promising, non‐invasive therapeutic alternative to stem cell injections. Integrating findings from our previous and current studies [8, 11], we demonstrate that coculture‐derived EVs enhance the activity of ACL cells, BMSCs, and hamstring tenocytes while concurrently reducing tenocyte apoptosis. This dual effect supports tenocyte survival and promotes graft maturation and integration. These cellular effects are particularly relevant to the early phases of graft ligamentization, during which apoptosis and reduced cellularity hinder remodeling. By improving tenocyte survival and migration, coculture‐derived EVs may help preserve cellularity during this vulnerable period and support more efficient graft maturation. Clinically, EV‐based augmentation could complement remnant‐preserving ACL reconstruction and may also be applied during early rehabilitation to counteract apoptosis during periods of heightened graft vulnerability. As a biologically based, non‐invasive strategy, EVs show strong potential to enhance tendon graft healing and improve outcomes after ACL reconstruction.

There are several limitations to this study. First, we focused on the effects of EVs on hamstring tenocytes in vitro; therefore, further validation in animal models is required to confirm their biological relevance in vivo. Second, although the precise mechanisms by which coculture‐derived EVs attenuate tenocyte apoptosis remain to be elucidated, our findings consistently demonstrate their superior protective effects compared with BMSC‐EVs. Third, this study did not include a vehicle‐ or PBS‐treated EV control, which limits our ability to fully exclude potential effects related to the isolation buffer or handling procedures. Finally, other paracrine factors present in the coculture medium, produced through interactions between ACL cells and BMSCs, were not characterized in this study. Nevertheless, within the scope of our experimental observations, these limitations do not compromise the main conclusion that coculture‐EVs exert enhanced pro‐regenerative and anti‐apoptotic effects on hamstring tenocytes.

In conclusion, this study demonstrates that coculture‐derived EVs significantly enhance hamstring tenocyte activity and reduce apoptosis compared with BMSC‐EV and non‐EV treatment groups. These findings highlight the potential of coculture‐EVs as a promising strategy for promoting tendon graft maturation and improving outcomes following ACL reconstruction. However, further research, including in vivo studies, clinical trials, and investigations into the underlying signaling mechanisms, is needed to fully elucidate the therapeutic potential and clinical application of EV‐based therapies in orthopedic surgery.

## Funding

This study was supported by grants from Kaohsiung Municipal Siaogang Hospital (H‐113‐01), the Ministry of Science and Technology, Taiwan (NSTC 114‐2314‐B‐037‐055‐MY3), and Regenerative Medicine and Cell Therapy Research Center, Kaohsiung Medical University, Taiwan (KMU‐TC114A02).

## Conflicts of Interest

The authors declare no conflicts of interest.

## Data Availability

The data that support the findings of this study are available from the corresponding author upon reasonable request.
